# Supplementary material for: Local deprivation predicts right-wing hate crime in England
Source: PLoS One. 2023 Sep 6;18(9):e0289423. doi: 10.1371/journal.pone.0289423 (PMC10482284; doi:10.1371/journal.pone.0289423)
Supplement: S1 Appendix — (DOCX) [file pone.0289423.s001.docx]

**Supplementary Appendix for “Local deprivation predicts right-wing hate crime in England”**

**Data**

In this section we describe in greater detail the data that we use on racially and ethnically motivated hate crimes and local deprivation in England. Our unit of analysis is the neighborhood, measured as Lower Super Output Areas (LSOAs).

*Outcome: Hate crimes*

We use data on racially and ethnically motivated hate crimes recorded by the UK Police for 2015-2021 as realizations of right-wing extremist violence to proxy the level of right-wing radicalization across neighborhoods in England. We use the logged cumulative number of hate crimes across English LSOAs between 2015 and 2018 (regression and training sample) and between 2019 and 2021 (hold out period). We divide the cumulative number of hate crime and divide by LSOA population size (in 100,000) to obtain our measure of right wing hate crime per capita rates.

*Measuring local deprivation*

To capture local deprivation, we use data for three sub-domains in the 2015 *English Indices of Deprivation* (IoD) [1] - Living Environment Deprivation, Education Deprivation, and Income Deprivation. IoD measure relative levels of deprivation in 32,844 neighborhoods in England. The indicators used to build the 2015 IoD version are based on a wide variety of data gathered between 2012/13. They thus predate the local level of right wing hate crimes examines in our analyses. Data are sourced from local, regional, and national administrative bodies with privileged data access, including HM Revenue and Customs, the Department for Transport, the Home Office, the Higher Education Statistics Agency, the National Health Service, and the HM Inspectorate of Constabulary.

Living Environment Deprivation is intended to capture fairness of distribution, environmental hazards, and procedural justice, which addresses whether people enjoy equal protection from hazards, and an equal opportunity to meaningfully affect policies and decisions about their environment [2]. The Living Environment Deprivation index is composed of a measure of “outdoors” living environment conditions, measuring air quality and road traffic accidents, and “indoors” living environment conditions measuring the quality of housing, including the existence of central heating and other basic home standards. Including the housing conditions in our measure of social deprivation is important to account for individuals’ capacity to moderate the hazards deriving from institutional failures in their most proximate environment.

“Education Deprivation” measures the lack of attainment and skills in the local population. The indicators fall into two sub-domains: one relating to children and young people and one relating to adult skills. These two subdomains are designed to reflect the “flow” and “stock” of educational disadvantage. That is, the “children and young people” sub-domain measures the attainment of qualifications and associated measures (“flow”), while the “skills” sub-domain measures the lack of qualifications in the resident working age adult population (“stock”). The indicators used to calculate the “children and young people” sub-domain include a. the average points score of pupils taking reading, writing and mathematics Key Stage 2 exams, b. the average capped points score of pupils taking Key Stage 4, c. the proportion of authorized and unauthorized absences from secondary school, d. the proportion of young people not staying on in school or non-advanced education above age 16, e. a measure of young people aged under 21 not entering higher education. The indicators used to calculate the “adult skills” sub-domain include a. the proportion of working-age adults with no or low qualifications, and b. the proportion of working-age adults who cannot speak English or cannot speak English well.

We conceptualize economic deprivation as individuals’ incapacity to independently satisfy the day-to-day living needs of themselves and their families. We use the “Income Deprivation” measure to proxy economic deprivation, since this measure captures the proportion of the population in a neighborhood experiencing deprivation relating to low income. IoD’ s definition of low income includes people that are out-of-work and those that are in work but who have low earnings. A combined count of income deprived individuals per neighborhoods is calculated by summing the following seven non-overlapping categories:

1. Adults and children in Income Support families,
2. Adults and children in income-based Jobseeker’s Allowance families,
3. Adults and children in income-based Employment and Support Allowance families,
4. Adults and children in Pension Credit (Guarantee) families,
5. Adults and children in Working Tax Credit and Child Tax Credit families not already counted, that is those who are not in receipt of Income Support, income-based Jobseeker’s Allowance, income-based Employment and Support Allowance or Pension Credit (Guarantee) and whose equivalized income (excluding housing benefit) is below 60 per cent of the median before housing costs,
6. Adults and children in Universal Credit families where no adult is in 'Working - no requirements' conditionality regime,
7. Asylum seekers in England in receipt of subsistence support, accommodation support, or both.

Across all three domains of deprivation, we use IoD scores. Compared to IoD ranks or deciles, scores allow for a weighted relative comparison between neighborhoods based on the proportion of the population affected by a particular kind of deprivation. Overall, lower score values refer to less deprivation in a neighborhood and higher score values refer to more deprivation. The income deprivation score ranges from 0 to 1, and the education and environment deprivation scores range from 0 to 100. All three distributions are right skewed, with most neighborhoods in England and Wales situated in the lower quantile of the ranges of the three deprivation measures. Descriptive visualizations of each measure are provided provided in the manuscript.

*Correlation matrix of deprivation measures*

|  | Income depr. | Living environment depr. | Education depr. |
| --- | --- | --- | --- |
| Income depr. | 1 | 0.255 | 0.835 |
| Living environment depr. |  | 1 | 0.105 |
| Education depr. |  |  | 1 |

*Other covariates*

We draw on 2011 UK census data [3] to include several neighborhood-level demographic and socio-economic covariates in our analysis to provide a null benchmark and, in the case of the regression models, to reduce confounding.

We include total population size (logged), average age, percent of rural/urban population, level of unemployment, the Black and Minority Ethnic (BAME) population share, the share of people of Christian religion, and the share of people of Arab ethnicity. We also include police force district fixed effects to partial out differences between districts. Police force districts are part of the FOI data provided by the Home Office’ Crime Analysis Unit, as referenced in the manuscript.

Table S1: Full list of police force districts and the number of neighborhoods contained

|  | Police force district | Number of LSOAs |
| --- | --- | --- |
| 1 | Avon & Somerset | 1005 |
| 2 | Bedfordshire | 381 |
| 3 | Cambridgeshire | 487 |
| 4 | Cheshire | 652 |
| 5 | Cleveland | 352 |
| 6 | Cumbria | 321 |
| 7 | Devon & Cornwall | 1034 |
| 8 | Dorset | 452 |
| 9 | Durham | 389 |
| 10 | Dyfed-Powys | 308 |
| 11 | Essex | 1077 |
| 12 | Gloucestershire | 373 |
| 13 | Greater Manchester | 1673 |
| 14 | Gwent | 368 |
| 15 | Hampshire | 1194 |
| 16 | Hertfordshire | 690 |
| 17 | Humberside | 583 |
| 18 | Kent | 1065 |
| 19 | Lancashire | 941 |
| 20 | Leicestershire | 611 |
| 21 | Lincolnshire | 420 |
| 22 | London, City of | 6 |
| 23 | Merseyside | 910 |
| 24 | Metropolitan Police | 4829 |
| 25 | Norfolk | 538 |
| 26 | North Wales | 423 |
| 27 | North Yorkshire | 493 |
| 28 | Northamptonshire | 422 |
| 29 | Northumbria | 916 |
| 30 | Nottinghamshire | 679 |
| 31 | South Wales | 810 |
| 32 | South Yorkshire | 853 |
| 33 | Staffordshire | 687 |
| 34 | Suffolk | 441 |
| 35 | Surrey | 709 |
| 36 | Sussex | 999 |
| 37 | Thames Valley | 1415 |
| 38 | Warwickshire | 339 |
| 39 | West Mercia | 781 |
| 40 | West Midlands | 1680 |
| 41 | West Yorkshire | 1388 |
| 42 | Wiltshire | 417 |

**Robustness**

*Incomplete data on hate crime*

Some areas report zero hate crimes for the entire observation period (see visualization in the manuscript). Inspecting the temporal and spatial distribution of hate crime, the number of areas that report zero hate crimes suggests a structural break hinting at an alternative data generating process (DGP). Based on this observation, we think it likely that some areas simply fail to accurately report actual hate crime statistics to the Home Office.

To test whether this data distortion may infringe on our findings, we conducted two additional analysis steps: one in which we treat all areas that reported zero hate crime as missing and excluded them from the analysis (see Table A1), and one in which we explicitly modelled them as a separate DGP in a zero-inflated negative binomial (ZINB) regression (see Table A2). We find that removing areas with zero reported hate crimes, either completely or by moving them into a separate model stage, generally does not change the analyses’ implications. The ZINB models, albeit tending to overfit, provide clues about the nature of the zero cases: the first-stage regressions (logit models) suggest that areas which report zero hate crimes tend to score much higher across the deprivation scores than areas that report any number of hate crimes. In other words, and relating back to our initial conjecture, it seems to be especially areas with high levels of deprivation that tend to report incomplete hate crime statistics. If correct, this would lead to a downward bias in our main results.

Table S2: Linear regression, omitting neighborhoods with zero hate crime

|  | **Model 1** | **Model 2** | **Model 3** |
| --- | --- | --- | --- |
| Income deprivation | 2.247 |  |  |
|  | (0.109) |  |  |
| Living envir. deprivation |  | 0.016 |  |
|  |  | (0.001) |  |
| Education deprivation |  |  | 0.003 |
|  |  |  | (0.001) |
| Income depr. (neighb.) | 1.142 |  |  |
|  | (0.104) |  |  |
| Living envir. depr. (neighb.) |  | 0.001 |  |
|  |  | (0.001) |  |
| Education depr. (neighb.) |  |  | 0.006 |
|  |  |  | (0.001) |
| Covariates | x | x | x |
| Police district FE | x | x | x |
| Num.Obs. | 26356 | 26356 | 26356 |
| R2 | 0.383 | 0.405 | 0.370 |
| RMSE | 0.81 | 0.80 | 0.82 |

Table S3: Zero-inflated negative binomial (ZINB) regression of hate crime rate

|  | **Model 1** | **Model 2** | **Model 3** |
| --- | --- | --- | --- |
| **Count model (NB, 2^nd^ stage)** |  |  |  |
| Income deprivation | 1.516 |  |  |
|  | (0.111) |  |  |
| Living envir. deprivation |  | 0.020 |  |
|  |  | (0.001) |  |
| Education deprivation |  |  | 0.000 |
|  |  |  | (0.001) |
| Income depr. (neighb.) | 1.026 |  |  |
|  | (0.107) |  |  |
| Living envir. depr. (neighb.) |  | 0.002 |  |
|  |  | (0.001) |  |
| Education depr. (neighb.) |  |  | 0.007 |
|  |  |  | (0.001) |
| Covariates | x | x | x |
| Police district FE | x | x | x |
|  |  |  |  |
|  |  |  |  |
| **Zero model (logit, 1^st^ stage)** |  |  |  |
| Income deprivation | −7.985 |  |  |
|  | (0.530) |  |  |
| Living envir. deprivation |  | −0.017 |  |
|  |  | (0.002) |  |
| Education deprivation |  |  | −0.016 |
|  |  |  | (0.002) |
| Income depr. (neighb.) | −2.796 |  |  |
|  | (0.443) |  |  |
| Living envir. depr. (neighb.) |  | 0.005 |  |
|  |  | (0.003) |  |
| Education depr. (neighb.) |  |  | −0.010 |
|  |  |  | (0.002) |
| Covariates | x | x | x |
| Police district FE | x | x | x |
| Num.Obs. | 32201 | 32201 | 32201 |

*Conditional effects of deprivation*

The degree to which local deprivation influences radicalisation depends on individuals’ subjective perceptions of how deprived they are. While the measurable levels of deprivation serve as our baseline, people’s subjective awareness likely deviates from this objective baseline due to various factors. Especially for populations that are already vulnerable, their sense of identification with their local community may matter. To this extent, in Table S3 we assess whether the effect of local deprivation on hate crime systematically varies across ethnic diversity strata. We find that our original results stay robust to this change in model specification. We also find that the effects of living environment deprivation and education deprivation are not moderated by ethnic diversity, while the effect of income deprivation on hate crime decreases with higher levels of ethnic diversity.

*Cluster-robust standard errors*

While there are no strong theoretical expectations for the cluster-specific heteroskedasticity, we re-estimate our main models with cluster-robust standard errors at the level of police districts in Table S4. Our inferences remain the same.

Table S4: Main models with ethnic diversity interaction

|  | **Model 1** | **Model 2** | **Model 3** |
| --- | --- | --- | --- |
| Income deprivation | 5.773 |  |  |
|  | (0.194) |  |  |
| Living envir. deprivation |  | 0.023 |  |
|  |  | (0.001) |  |
| Education deprivation |  |  | 0.013 |
|  |  |  | (0.001) |
| Income depr. (neighb.) | 1.791 |  |  |
|  | (0.169) |  |  |
| Living envir. depr. (neighb.) |  | -0.001 |  |
|  |  | (0.001) |  |
| Education depr. (neighb.) |  |  | 0.009 |
|  |  |  | (0.001) |
| Income depr. * BAME | -0.108 |  |  |
|  | (0.005) |  |  |
| Living envir. depr. * BAME |  | 0.000 |  |
|  |  | (0.000) |  |
| Education depr. * BAME |  |  | 0.000 |
|  |  |  | (0.000) |
| BAME perct. | 0.020 | 0.005 | 0.008 |
|  | (0.002) | (0.001) | (0.001) |
| Covariates | x | x | x |
| Police district FE | x | x | x |
| Num.Obs. | 32201 | 32201 | 32201 |
| R2 | 0.448 | 0.441 | 0.434 |

Table S5: Main models with cluster-robust standard errors

|  | **Model 1** | **Model 2** | **Model 3** |
| --- | --- | --- | --- |
| Income deprivation | 4.048 |  |  |
|  | (0.319) |  |  |
| Living envir. deprivation |  | 0.020 |  |
|  |  | (0.002) |  |
| Education deprivation |  |  | 0.008 |
|  |  |  | (0.001) |
| Income depr. (neighb.) | 1.704 |  |  |
|  | (0.268) |  |  |
| Living envir. depr. (neighb.) |  | -0.002 |  |
|  |  | (0.002) |  |
| Education depr. (neighb.) |  |  | 0.008 |
|  |  |  | (0.002) |
| Covariates | x | x | x |
| Police district FE | x | x | x |
| Num.Obs. | 32201 | 32201 | 32201 |

References

[1]. UK Department for Communities and Local Government. English Indices of Deprivation https://www.gov.uk/government/statistics/english-indices-of-deprivation-2015

[2] Mitchell, G., Norman, P., Mullin, K.. Who benefits from environmental policy? An environmental justice analysis of air quality change in Britain, 2001–2011. Environmental Research Letters. 2015; 105009, DOI 10.1088/1748-9326/10/10/105009

[3] Office for National Statistics, 2011. Census in England and Wales. Retrieved from: https://www.ons.gov.uk/census/2011census.
