# Supplementary material for: Local deprivation predicts right-wing hate crime in England
Source: PLoS One. 2023 Sep 6;18(9):e0289423. doi: 10.1371/journal.pone.0289423 (PMC10482284; doi:10.1371/journal.pone.0289423)
Supplement: Attachment — Submitted filename: 2023-07-14_PLOSONE_resubmission_memo.docx [file pone.0289423.s002.docx]

**Revision Memo: PONE-D-23-08904 "Local deprivation predicts right-wing hate crime in England"**

We refer to your email of 12 June regarding our submission PONE-D-23-08904 "Local deprivation predicts right-wing hate crime in England". We are very grateful for the invitation to resubmit a revised version, and we appreciate the efforts of the editor and reviewers for engaging so thoroughly with the manuscript.

We respond below to the main concerns raised, ordered by topic, with our responses and comments in italics. The reviewers also made a number of smaller comments and suggestions that we have tried to incorporate to the best of our ability in the revisions. We believe the revised version has been notably strengthened as a result of the revisions made in response to the comments.

Please do not hesitate to contact us if we can help with any additional information.

Sincerely yours,

The authors

**A. Theory**

**A1) Clarification of relationship between community deprivation and attacks**

R1: “If individuals indeed strongly identify with their local community, it is unclear why one would expect that observing deprivation within one’s own community would lead to an increase in far-right hate crime within that same community. …. Currently, this assumption is not explicitly discussed …. I believe the paper would benefit from making this assumption more explicit and from strengthening the theoretical arguments and references to empirical work to back it up”. On a related issue, R2 warns against “framing individual-level research as the ‘wrong unit of analysis’ … and [how] these kinds of dichotomies can often be a fallacy

*We have expanded our discussion of why we would expect to find a relationship between local deprivation and attacks. In brief, we argue that hate crimes typically target specific communities or individuals based on their characteristics. There is empirical evidence demonstrating that perpetrators of hate crimes tend to carry out violence within their local neighborhood. There are a number of reasons why perpetrators often seek out victims within their immediate vicinity, notably 1) it is easier to identify targets they hold prejudice against in the local community, 2) access, planning and execution is typically easier for local targets, 3) local targets often have symbolic significance for both perpetrators and targeted communities, and 4) local hate crimes will often receive more attention in the immediate community, which in turn can be helpful for generating fear, exerting control, and recruiting to the cause. We agree with R2 that it is unhelpful to draw a false dichotomy between individual level analysis and other levels of aggregation. We have revised the text to emphasize more clearly the added and complementary value of local level analysis.*

**A2) Relationship to relative and absolute deprivation.**

*R2 asked us to clarify the relationship between relative and absolute deprivation in the theoretical framework. We appreciate that our previous discussion may have been confusing on this point, and we have clarified our conceptualization and actual measures of deprivation in the manuscript. We emphasize that what we think drives the key relevant comparisons here are differences between neighbourhoods, not within community differences*.

**A3) Discussion of UK prevent scheme**

R2 notes that … “This scheme looks to me as an under simplification of a complex issue … the scheme implies that a productive path for prevent terrorism is to scrutinize vulnerable groups and, most specifically, individuals who sympathize with a certain ideology. … What would be the outcome of leveraging a group’s vulnerability and political attitudes to identify terrorism? Wouldn’t we simply run the risk of add burden to these vulnerabilities and attitudes?”
*We appreciate the comments on the UK Prevent scheme. We also share many of the concerns expressed over the scheme, and we try to be more upfront about this in the manuscript. At the same time, this scheme has clearly guided policy and discussion, and as such it is useful to discuss our approach in comparison to this. We did not intend to apply the term “vulnerable” as a value judgement or to underplay individual agency, rather we simply seek to examine if features discussed in this context can help identify local contexts with a higher probability of radicalization and observed attacks. We primarily discuss the potential influence of deprivation in our manuscript rather than measures against potentially radicalized individuals or communities. We have revised the discussion so as to be much clearer on this and avoid potential misunderstanding.*

**A4) Diffusion and network effects**

The editor noted that “it would be valuable … to explore the possibility that the local deprivation effect can be understood within the context of micro-dynamic models that incorporate social networks as a mechanism for the diffusion of civil unrest behavior … [discussing and referencing] Braha (2012) … would provide additional insight into the dynamics of local deprivation and hate crime behavior.

*We entirely agree that networks and diffusion are important components in accounting for observed unrest and hate crime. We have added a brief discussion and referenced work on the diffusion of political violence. However, a full examination of this would require a much more complex analyses of the role of “local fundamentals” vs “position”, and ideally consider detailed analyses of individual connections using actual data rather than proxies based on geographical distance. We also note that very strong diffusion or emulation effects could plausibly attenuate the direct local relationships, and this appears to be the case in many studies of organized political violence.*

**A5) Time ordering in exposure and response**

R1 and R2 noted that gap in time between deprivation indices for 2012-2013 and hate crimes committed 2015-2019. “… would [it] be possible to say something about the timing between witnessing deprivation and committing hate crimes? E.g., is there any theoretical work suggesting what time frame matters, how long one should be exposed to deprivation before moving to committing hate crimes?” (R1)

*We use prior deprivation data since we want to ensure that the deprivation data is clearly prior and is not likely to arise from as a result of or influenced by our outcome. There is little direct guidance in previous work on the likely timing of exposure and potential impact on attacks, but we believe the lag length in our case is a reasonable length to reflect the impact of past exposure to deprivation. In practice, there exists considerable persistence in deprivation over time, and this is supported by the data on deprivation published by Ministry of Housing (see for example Indices of Deprivation: 2019-2015 at https://dclgapps.communities.gov.uk/imd/iod_index.html).* *We have revised our discussion so as to make clearer the assumptions guiding our analysis, the limits related to actually considering sensitivity to timing in the current data and our analysis, and the value of future more detailed research on this.*

**A6) Conditional effects**

R1 notes that “The extent to which individuals identify with their local neighborhood [might vary by] … ethnic diversity. … Would there be a way to leverage the heterogeneity in this variable (or other variables the authors may think of), e.g., looking at an interaction with the deprivation indices?”

*We think it is plausible that such heterogeneity may be present in the observed data, and we have conducted analyses where we interact ethnic diversity, proxied as the local population share with BAME background, with our indicators of deprivation. We find that ethnic diversity does not substantially moderate the effects of living environment deprivation and education deprivation, but that high levels of diversity may mitigate the effect of income deprivation. However, we ultimately feel that extending the analysis further along these lines is somewhat removed from our main focus in the manuscript, and we thus report in an appendix without an extended discussion in the main manuscript text. We think a fuller investigation of this would amount to a different project and better pursued in a separate manuscript. We also note that measuring conditional interactions or moderators reliably demands a great deal more of the data than main effects, and could benefit from more extended data than we have at hand now.*

**B. Data and empirical analysis**

**B1) Lagged dependent variables and autoregressive trends**

The editor noted that “to further enhance its robustness, I would suggest that the authors incorporate lagged dependent variables in the regression model …[to] account for the likelihood that previous instances of hate crimes contribute to subsequent hate crimes, potentially overshadowing the effect of local deprivation”.

*We appreciate the point made, and we entirely agree that hate crime data are likely to show considerable persistence and autoregressive trends. Including a lagged dependent variable would be natural if we had panel data, but unfortunately, we do not – the statistics for hate crimes that we rely on, and the time gaps between releases of the Indicators of Deprivation, do not allow for a time-series setup. Therefore, we are essentially only able to conduct a cross-sectional analysis, and it is not possible to include a lagged dependent variable in the regression model. We now explicitly clarify this in the revised version of the manuscript. We also do not propose to measure feature/variable importance by contribution to fit in-sample, which is prone to overfitting, but rather contribution to out-of-sample prediction (see B3 below).*

**B2) Measures and specific components**

R1 notes that the income deprivation index [includes] ‘Asylum seekers in England in receipt of subsistence support, accommodation support, or both’. I wonder to what extent this indicator is driving the estimated coefficients on the income deprivation index … It would be interesting to see a robustness check in which this indicator is dropped from the income deprivation index (and perhaps included as a covariate).”

*We have looked at the* *available data and the Home Office statistics to verify if we could find any plausible proxies. There is unfortunately no publicly available sub-national data, so we are unable to probe how sensitive the findings may be to this. We believe it is likely that the IoD obtained these data directly from the Home Office at LSOA level. The only way which we might be able to obtain these data would be to submit a FOI request, which may or may not be successful. This is not feasible within the deadline for the resubmission, and we feel that this goes beyond the main priorities for the resubmission. The fact that we consider alternative indicators for deprivation other than income, which do not include asylum seekers in receipt of support, makes it unlikely that this component alone would dominate the results. However, we now acknowledge this limitation in the main text and explicitly flag this as an area for future study.*

On a similar note, R3 argues that the status of the deprivation measures must be clarified, and that we should “consider … average[ing] them … or evaluate models containing more than one of them at a time”

*We now report the correlations in the Supplementary Appendix. We understand the case for trying to aggregate the information into a combined deprivation index, but to do this systematically should ideally be based on the raw inputs of the indices, rather than averaging across aggregate indices with different scales/metrics. Ultimately the regression models are all in-sample, and they are intended to evaluate the conditional associations/causal effect approximations in a simple manner. For this particular purpose, including all deprivation measures in one model would make the interpretation more difficult. For prediction, on the other hand, we actually use a random forest model that includes all the deprivation scores in the same models for computing the predictions of future levels of hate crime. We have now clarified this issue in the discussion in the manuscript.*

**B3) Role of prediction in model evaluation**

*Rs 1 and 2 raised some question about the value of splitting the data, emphasis on prediction, and added value of the model with deprivation. We have now revised the text so as to clarify why cross validation is an important check on overfitting to observed data. It also helps emulate the potential to use measures for out of sample forecasts. We have also clarified that even if the variance accounted for may seem small in absolute terms, there is a near double increase for the deprivation model. We think this demonstrates the added value, although we try to be explicit on not overstating the absolute predictive power.*

**B4) Clarify data**

*Rs1, 2, and 3 asked for clarification for many aspects of the data, including the timing of the covariates, and the size/number of police force districts used for fixed effects and relationship to local LSOAs, and the district reporting no hate crimes. We have revised our discussion so as to provide more details explicitly on the issues raised. We also discuss in more detail the potential bias from underreporting of hate crimes (which under plausible condition would likely attenuate the reported relationship with deprivation). The Home Office informed us about the police districts that did not submit any reports in specific years (where the “zero hate crimes” is the result of a reporting issue), and these districts were removed from all analyses to begin with. In addition, we report results with all units with zero hate crimes removed from the analyses in the Supplementary Appendix, referenced in the main manuscript.*

**B4) Clarify the analyses reported**

*Rs 1,2, and 3 called for more details in explanatory notes (e.g., types of standard errors), and we have implemented these in the revised manuscript. We also report AICs in addition to R2, as requested.*

**B5) Figures/image resolution**

*R3 suggested to revise Figure 6 to include two side-by-side panels, one for the without deprivation model, and one for the with deprivation, with a best fitting regression line. We have also improved the resolution of the images in the manuscript. The small font sizes are the result of downscaling in MS Word and potentially only PDF conversion. This will not be an issue in the published version of the manuscript using the separate images.*

**B6) Supplementary analyses**

*R1 suggested a robustness check relying on the ACLED data. We appreciate this suggestion, but we are unable to use ACLED, since the ACLED data for the United Kingdom are limited to the period between 2020 and 2022, cutting significantly our sample. (Moreover, ACLED has a restrictive terms of use license, which does not allow for comparisons to other data sources, see* [*https://acleddata.com/acleddatanew/wp-content/uploads/2022/06/ACLED-Terms-of-Use-Attribution-Policy_V2_8June2022.pdf*](https://acleddata.com/acleddatanew/wp-content/uploads/2022/06/ACLED-Terms-of-Use-Attribution-Policy_V2_8June2022.pdf)*). In general, with news-based event data (like ACLED and GTD) we can expect the coverage of the events to be more limited and arguably thus inferior to our administrative data. We believe the selection problems from media reports are likely to be worse than the potential problems arising from differences in reporting by administrative agencies. We discuss in more detail how to best attempt to mitigate issues arising from underreporting.*

**C. Broader implications/policy**

*Some of the reviewers raised issues about the potential broader implications and actionable policies that our manuscript could be seen as supporting. We have tried to be careful to be modest in our discussion of policy implications, in particular since we have not studied intervention to reduce deprivation or inequality. However, we note the reductions in child poverty under New Labour as a possible example of how reforms seeking to reduce inequality could have wider impacts or dividends on violence, even if reducing violent attacks by itself is the main target of policy proposals.*

**D. Journal Requirements:**

**D1) Compliance with style requirements, including file names.**

*We have carefully reviewed the guidelines to ensure that the manuscript and file names follow the PLOS One guidelines.*

**D2) Role of funders**

Please state what role the funders took in the study… Please include this amended Role of Funder statement in your cover letter; we will change the online submission form on your behalf.

*We have included the suggested text in our cover letter: "The funders had no role in study design, data collection and analysis, decision to publish, or preparation of the manuscript."*

**D3. Data Availability statement**

*We will update your Data Availability statement to reflect the information you provide in your cover letter.*

*We have made our data available on the Harvard Dataverse at https://doi.org/10.7910/DVN/GSRPQY.*

**D4) Re license/copyright for maps used for Figures 2 and 5** 
*These images have been generated by shape files that are publicly available from the UK Office for National Statistics. They are supplied under the Open Government Licence. We have added the source and license as “Office for National Statistics licensed under the Open Government Licence v.3.0”.*

**D5). … include captions for your Supporting Information files at the end of your manuscript, and update any in-text citations to match accordingly.**

*We have now implemented this.*
